# The application of a monolithic triphenylphosphine reagent for conducting Ramirez *gem*-dibromoolefination reactions in flow

**DOI:** 10.3762/bjoc.9.207

**Published:** 2013-09-02

**Authors:** Kimberley A Roper, Malcolm B Berry, Steven V Ley

**Affiliations:** 1 Innovative Technology Centre, Department of Chemistry, University of Cambridge, Lensfield Road, Cambridge, Cambridgeshire, CB2 1EW, U.K.; 2 GlaxoSmithKline, Gunnels Wood Road, Stevenage, Hertfordshire, SG1 2NY, U.K.

**Keywords:** bromination, flow chemistry, Ramirez *gem*-dibromoolefination reaction, solid-supported reagent, triphenylphosphine monolith

## Abstract

The application of a monolithic form of triphenylphosphine to the Ramirez *gem*-dibromoolefination reaction using flow chemistry techniques is reported. A variety of *gem*-dibromides were synthesised in high purity and excellent yield following only removal of solvent and no further off-line purification. It is also possible to perform the Appel reaction using the same monolith and the relationship between the mechanisms of the two reactions is discussed.

## Introduction

The advantages of applying flow chemistry processing to organic synthesis have been extensively demonstrated in the literature, increasing the safety, efficiency and reproducibility of many organic chemistry reactions, causing this technology to be accepted as an important new tool to aid the modern research chemist [1–7]. Combining this enabling technology with solid-supported reagents and scavengers offers synergistic benefits over using the two technologies independently. Utilising polymer-supported reagents and scavengers to purify the flow stream permits telescoping of reaction sequences or facilitates direct isolation of pure compounds from flow reactors, removing the need for labour-intensive manual operations [8–13]. Reagents are typically supported on low-crosslinked gel-type or macroporous beads; however, these are characterised by poor mass transfer properties as well as presenting practical problems when used in packed beds in flow reactions due to changes in structure and morphology when subjected to solvents of varying polarity [14–15]. To avoid some of the problems associated with using resin beads, monolithic supports have been developed for use in continuous-flow chemistry systems. Monoliths are a single continuous piece of uniformly porous material, prepared by precipitation polymerisation of a functionalised monomer [16–20]. The monolith internal structure varies compared to bead-like supports, consisting of a combination of large macropores for flow through passage, in combination with a network of smaller mesopores to allow diffusion towards the active sites. This combined geometry has been shown to result in superior chemical efficiency over traditional supports by providing a shorter diffusion pathway to active sites via convective flow-through the macropores, as well as providing lower void volumes [16]. Practically, their rigid structure is secure over a wide range of solvents and under reasonable pressures compared to beads due to a high degree of cross linking, making them advantageous when applied to flow processes [21–22].

Originally monoliths were developed to facilitate the isocratic separation of peptides [17,23]; however, our group and others have shown interest in using monolithic supports to facilitate key chemical transformations [24–35]. The above advantages of using monolithic supports over traditional beads in flow chemistry protocols can greatly facilitate the synthesis of fine chemicals using these enabling technologies [36]. We have recently reported on the development of a monolithic triphenylphosphine reagent and its application to the Staudinger aza-Wittig and Appel reactions in flow [37–40]. The immobilisation of triphenylphosphine in this manner allowed the facile production of a collection of pure compounds using flow chemistry technologies with no need for further offline purification. Following the successful application of this monolith to the Appel reaction (the transformation of alkyl alcohols to the corresponding bromides), we wished to investigate the application of this monolith to the closely related Ramirez *gem*-dibromoolefination reaction; the formation of *gem*-dibromoolefins from aldehydes or ketones.

**Scheme 1:**
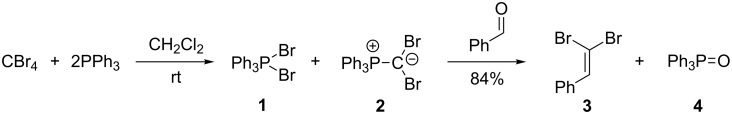
Formation of *gem*-dibromoolefin **3** from the reaction of carbon tetrabromide and triphenylphosphine as reported by Ramirez et al. [41].

In 1962 Ramirez, Desai and McKelvie reported the formation of dibromophosphorane **1** and (dibromomethylene)triphenylphosphorane (**2**) from the room temperature reaction of carbon tetrabromide with two equivalents of triphenylphosphine (Scheme 1) [41]. Addition of benzaldehyde then gave the desired *gem*-dibromoolefin, (2,2-dibromovinyl)benzene (**3**) in 84% yield. Triphenylphosphine oxide (**4**) was also isolated from the reaction as a byproduct. These *gem*-dibromoolefin products are particularly important intermediates in the one carbon homologation of an aldehyde into the corresponding terminal alkyne, known as the Corey–Fuchs reaction [42], and more recently stereospecific hydrogenolysis, Stille and Suzuki reactions have been used to further elaborate these useful products [43–45].

The triphenylphosphine oxide byproduct can often be difficult to remove from the reaction mixture, requiring extensive, time-consuming purification procedures to isolate the desired product in high purity. For Ramirez *gem*-dibromoolefination reactions, successful strategies have been developed to facilitate this separation through derivatising the triphenylphosphine (or its oxide) to achieve purification via filtration [46–47], as well as by immobilising the triphenylphosphine on a solid-support [48]. A polymer-supported equivalent of triphenylphosphine has also been successfully utilised by our group and by others in batch Wittig reactions [49–50], Mitsunobu and Staudinger aza-Wittig reactions [51–52], as well as many examples concerning the Appel reaction [51–57].

Following our success using a monolithic form of triphenylphosphine to facilitate the Appel reaction, we wanted to explore the use of this monolith for performing the Ramirez *gem*-dibromoolefination reaction in flow. The monolithic form of triphenylphosphine should have improved flow characteristics compared to bead-based equivalents circumventing the problems associated with using these solid-supported reagents in combination with flow techniques. Key intermediates for the Ramirez dibromoolefination reaction, **1** and **2** depicted in Scheme 1, are also known to be potential intermediates in the Appel reaction [58–59] and consequently we also wished to investigate the interplay between the two reaction mechanisms.

## Results and Discussion

### Formation of the triphenylphosphine monolith

**Scheme 2:**
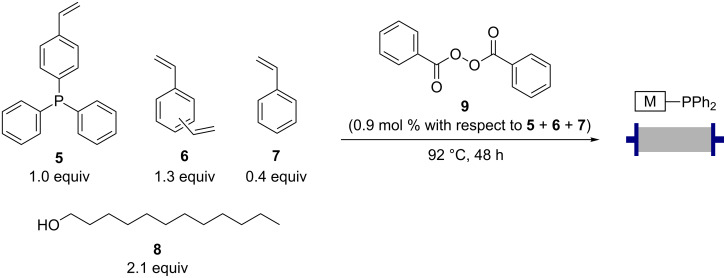
Formation of the triphenylphosphine monoliths.

The triphenylphosphine monoliths for the Ramirez reactions were formed using precipitation polymerisation of the phosphine monomer **5** (Scheme 2). A polymerisation mixture of the triphenylphosphine monomer **5**, cross-linking components divinylbenzene (**6**) and styrene (**7**) along with the porogen, 1-dodecanol (**8**), was heated to 50 °C until a homogeneous mixture was achieved. The initiator, dibenzoyl peroxide (**9**) was then added and the temperature maintained at 50 °C until this had completely dissolved. The mixture was then transferred to a glass column, the ends sealed with custom-made PTFE end pieces and heated to 92 °C for 48 hours using a Vapourtec R4 heating unit. This protocol can be clearly viewed in a video previously released by our group [40], however the Ramirez monoliths employ a higher ratio of styrene to divinylbenzene. This results in a lower proportion of crosslinking within the monolith, allowing greater flexibility in the backbone of the polymer whilst still maintaining desirable monolithic characteristics during flow reactions. This greater flexibility has previously been shown to assist with the formation of active species **1** and **2** in solid-supported triphenylphosphine beads, by allowing neighbouring group interactions between the triphenylphosphine residues.

**Figure 1:**
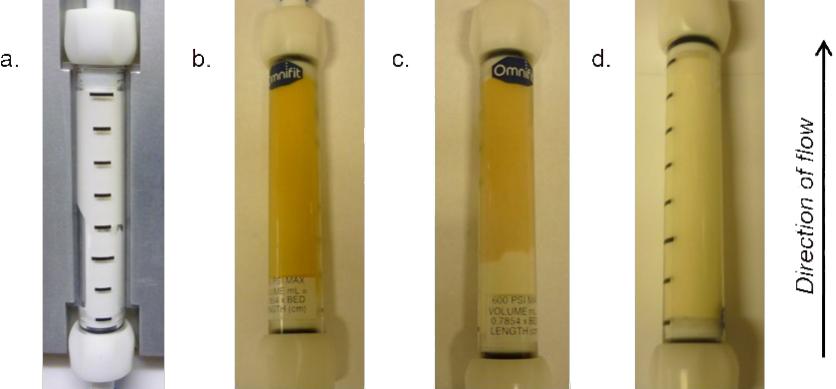
a. An unfunctionalised triphenylphosphine monolith; b. Monolith after functionalisation with carbon tetrabromide at 0 °C; c. Monolith after complete consumption of the active Ramirez *gem*-dibromoolefination species; d. Monolith after complete consumption of the active Ramirez *gem*-dibromoolefination species and the Appel brominating species.

The resultant white polymer (see Figure 1, a) was cooled to room temperature and the end plugs exchanged with standard flow-through end pieces. The porogen and any unreacted starting materials were then eluted from the monolith using a stream of dichloromethane at elevated temperature (60 °C). This polymerisation protocol consistently gave a low pressure drop across the monoliths for use in flow reactions. The monoliths were calculated to have a phosphorus loading of 1.85 mmol of phosphorus per gram, resulting in approximately 4.63 mmol of phosphorus per monolith.

### Loading the monolith to give the active Ramirez brominating species

**Scheme 3:**
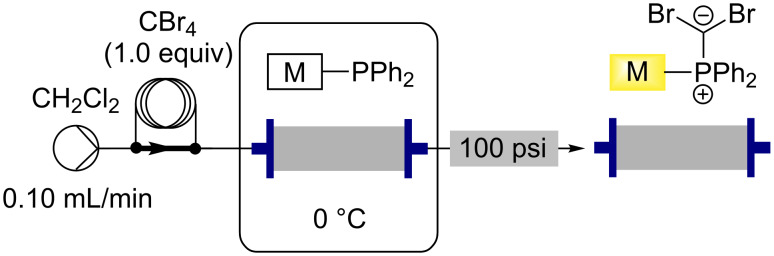
Functionalising the triphenylphosphine monolith to give the active Ramirez monolith using carbon tetrabromide.

Loading the monolith with carbon tetrabromide to give the active species for the Ramirez *gem*-dibroomolefination reactions was found to proceed in a facile manner using a single pass protocol with the monolith being cooled to 0 °C (Scheme 3). Cooling the monolith by submerging it in an ice-water bath was found to be necessary to prevent the formation of an inseparable side product, observed if reactions were performed at room temperature.

Interestingly, an external source of triphenylphosphine was not required to form the solid-supported equivalents of active species **1** and **2**, indicating that the polymer chains within the monolith have sufficient conformational freedom to allow neighbouring group interactions between triphenylphosphine sites. Any attempts to use a solution of triphenylphosphine to increase the active loading of the monolith was found to result in the formation of insoluble triphenylphosphine salts which crystallised and blocked the flow tubing downstream of the monolith.

The formation of the active species was accompanied by a colour change, resulting in a bright yellow polymer (Figure 1, b). Each monolith was shown to have an active loading towards the Ramirez transformation of approximately 0.8 mmol. Although this is a relatively low active loading, this is not unexpected as two equivalents of triphenylphosphine are required for the formation of one equivalent of the active Ramirez brominating species.

### Ramirez *gem*-dibromoolefination reactions in flow

**Scheme 4:**
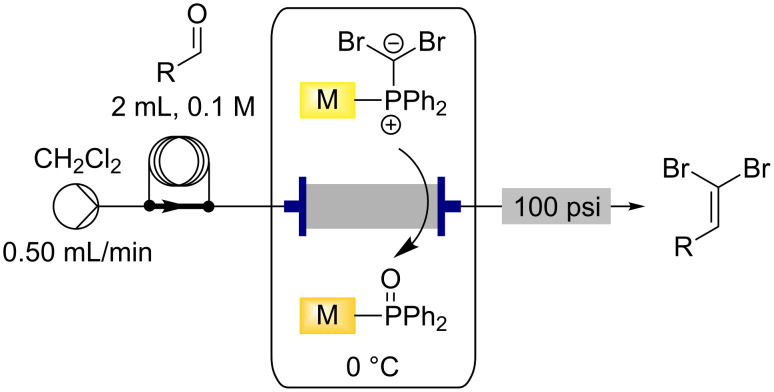
Flow synthesis of *gem*-dibromoolefins using the functionalised triphenylphosphine monolith.

With the functionalised monolith in hand, it was then used to perform the Ramirez *gem*-dibromoolefination reaction in flow to transform aldehydes into their corresponding *gem*-dibromoolefins. A 0.1 M solution of the aldehyde in dichloromethane was prepared and introduced into the flow system via the use of a sample loop. This solution was passed through the loaded monolith at a rate of 0.5 mL/min while the monolith was maintained at 0 °C using a cooling bath (Scheme 4). The output was collected for 1 h 15 min and the solvent removed in vacuo to give complete conversion to the pure *gem*-dibromoolefin product without any further manipulation.

This procedure was applied to a wide variety of aldehydes, giving the *gem*-dibromoolefin products in high yields and purity following only removal of the solvent by evaporation (Table 1).

**Table 1:** *Gem*-dibromides prepared from the corresponding aldehydes using the triphenylphosphine monolith in flow.

Entry	Starting material	Product	Isolated yield (%)^a^

1	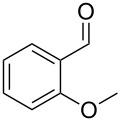	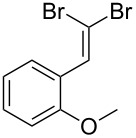	80
2	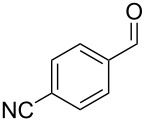	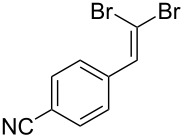	95
3	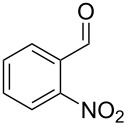	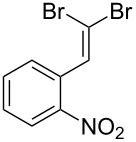	93
4	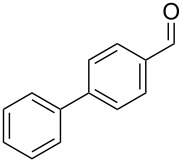	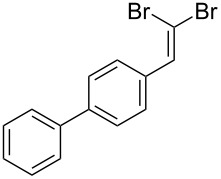	98
5	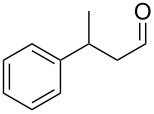	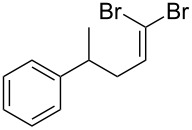	79
6	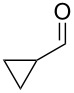	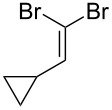	78^b^
7	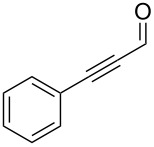	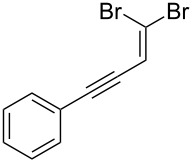	83
8	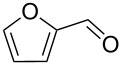	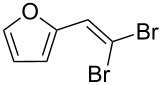	97
9	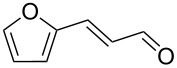	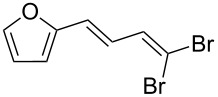	91
10	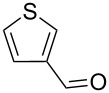	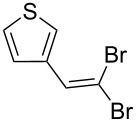	87
11	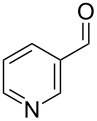	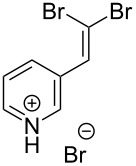	41^c^

^a^Reactions performed on a 0.2 mmol scale; ^b^product volatile, ^c^output collected for 2 hours rather than 1 h 15 min.

Benzylic aldehydes containing electron withdrawing and donating groups on the phenyl ring (Table 1, entries 1–4) were transformed in high yield, as well as alkyl aldehydes (Table 1, entries 5 and 6). Unsurprisingly, aldehydes containing a phenol moiety were found to give little or no mass return as the phenolic hydroxy group reacted with the triphenylphosphine sites within the monolith, leaving the product bound to the polymer. Interestingly the batch bromination of 3-phenylpropiolaldehyde (Table 1, entry 7) requires the addition of 2.5 equivalents of 2,6-lutidine [60], however pleasingly, this was not required when the substrate was brominated using the flow procedure. It was also possible to use the monolith on a series of heterocyclic substrates with high yields (Table 1, entries 8–10). However, nicotinaldehyde (Table 1, entry 11) was found to give a reduced yield and unusually contamination of subsequent products formed using the same monolith was observed. X-ray crystallography and mass spectrometry confirmed that the product isolated was the hydrobromide salt of the desired *gem*-dibromoolefin, presumably formed from an additional reaction with the monolith. The salt formed will coordinate to other ionic sites within the monolith, reducing the isolated yield and resulting in contamination of further products as it is slowly released from the column.

A colour change was associated with the reaction, with the monolith changing from a bright yellow to dull dark yellow colour (Figure 1, b and c). A single monolith could be used for multiple transformations with no cross contamination between substrates run in sequential reactions through a single monolith (with the exception of the nicotinaldehyde substrate explained above).

An important test of this methodology was the application to α-chiral aldehydes, to ensure that racemisation of the sensitive chiral centre is avoided in chiral structures (Table 2). A butane-2,3-diacetal derived aldehyde (Table 2, entry 1) and a diastereomeric aldehyde containing an acetonide (Table 2, entry 2) were successfully brominated using the flow protocol, being isolated in high yield with retention of stereochemistry as determined by ^1^H NMR. The method was then applied to an enantiopure aldehyde (Table 2, entry 3) which could be transformed to the desired product in high yield [61].

**Table 2:** α-Chiral aldehydes and ketones containing electron-withdrawing groups converted to the corresponding *gem*-dibromides using the triphenylphosphine monolith in flow.

Entry	Starting material	Product	Isolated yield (%)^a^

1	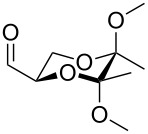	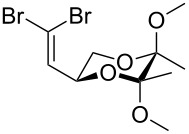	95
2	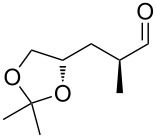	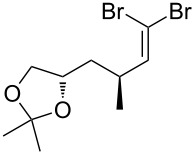	84
3	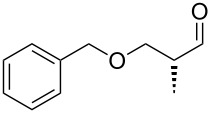	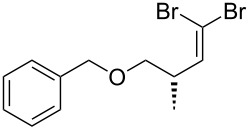	91
4	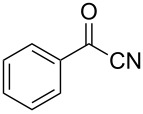	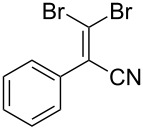	98
5	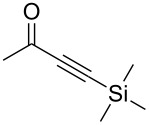	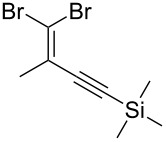	84^b^

^a^Reactions performed on a 0.2 mmol scale; ^b^reaction run at 0.10 mL/min with a previously unused monolith.

There is also precedent for performing Ramirez *gem*-dibromoolefin reactions on carbonyl groups other than aldehydes, such as certain ketones activated using electron withdrawing groups [47]. A selection of these ketones were therefore subjected to the flow Ramirez reaction conditions (Table 2). Unsurprisingly, unactivated ketones such as cyclohexanone and benzophenone gave no conversion to the desired dibromide using the standard conditions. However, with some optimisation, an acyl cyanide (Table 2, entry 4) and a silyl protected ynone (Table 2, entry 5) could be converted to the desired *gem*-dibromoolefins respectively in high yields. Interestingly, it was found that full conversion could only be achieved for the silyl protected ynone using a low flow rate and a previously unused monolith, indicating some reduction in reactivity with each use of the monolith.

### Utilising the loaded monolith for the Appel reaction in flow

**Scheme 5:**
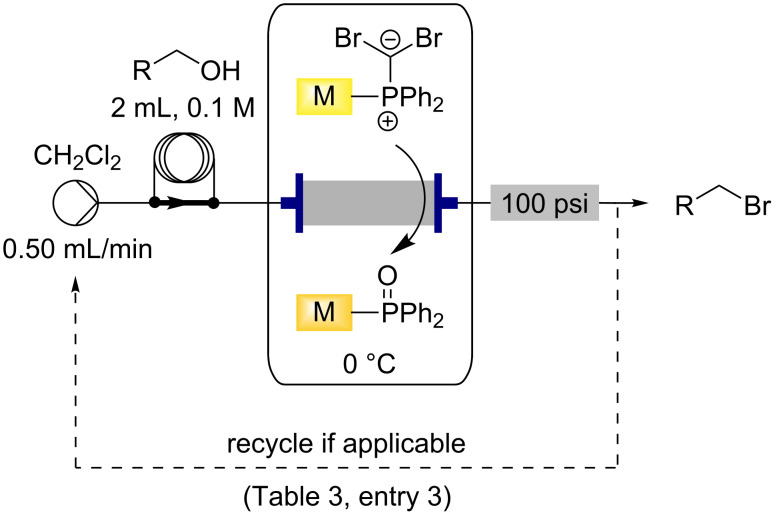
Flow synthesis of bromides from the corresponding alcohols using the functionalised triphenylphosphine monolith in the Appel reaction at 0 °C.

The two active species formed during the Ramirez *gem*-dibromoolefination reaction (**1** and **2** in Scheme 1) are also known to be potential intermediates in the Appel reaction and we have previously shown that these monoliths can facilitate this formation using similar conditions [39]. We wished to investigate the relationship between the two reactions and hoped to establish conditions to perform both reactions using a single protocol. Using a similar configuration to the Ramirez reactions in flow, a selection of alcohols were directed through the monolith loaded with carbon tetrabromide at 0 °C (Scheme 5). Gratifyingly it was found that the monoliths prepared for the Ramirez *gem*-dibromoolefination reactions could be used directly for the Appel transformation, giving the bromide products in high yield and high purity following removal of the dichloromethane solvent (Table 3). Citronellol (Table 3, entry 1) and an indole derived alcohol (Table 3, entry 2), could be transformed in a facile manner using a single pass of the alcohol through the monolith at 0 °C, however the allyl alcohol (Table 3, entry 3) required recycling through the monolith to effect complete conversion. In batch, this reaction required low temperature conditions (−78 °C) and the presence of base to give an isolated yield of 78% [62], however this could be improved to 90% by performing this reaction in flow at 0 °C. Loading the monolith using the protocol described above was found to give an approximate active loading of 0.6 mmol for the Appel reaction.

**Table 3:** Alkyl bromides prepared from the corresponding alcohols using the triphenylphosphine monolith for the Appel reaction in flow.

Entry	Starting material	Product	Conversion after one pass (%)^a^	Time required for full conversion^b^	Isolated yield (%)^c^

1	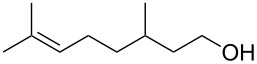	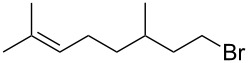	100	–	82
2	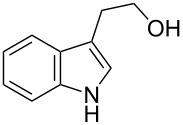	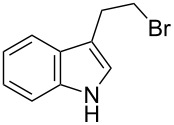	100	–	95
3	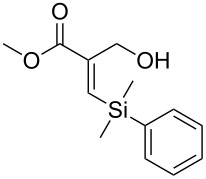	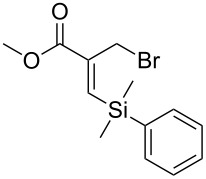	13	14 h 30 min	90

^a^One pass through the monolith at 0.5 mL/min, percentage conversion determined by ^1^H NMR analysis; ^b^substrate recirculated through the monolith at 0.5 mL/min until full consumption of starting material indicated by TLC; ^c^reactions performed on a 0.2 mmol scale.

**Scheme 6:**
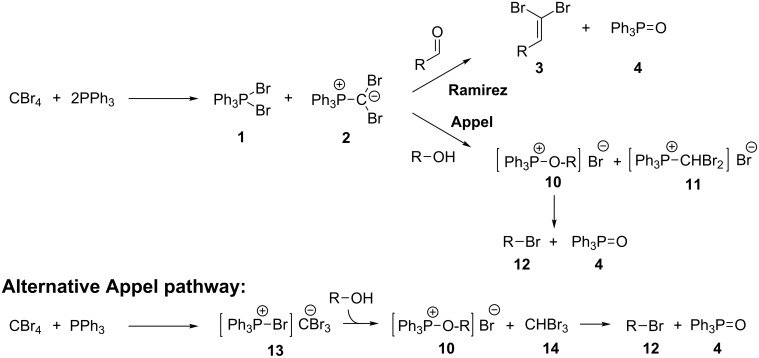
Mechanisms for the Ramirez and Appel reactions [41,59].

Utilising one monolith for both reactions potentially broadens the synthetic utility of the supported reagent and so performing both reactions sequentially using a single monolith was investigated. It was anticipated that these studies into the interplay between the reactions might also assist to elucidate the mechanism through which the Appel reaction proceeds on solid-support. It is known that the Appel reaction can proceed either through intermediates **1** and **2** which are common to both the Ramirez and Appel reactions, or via the alternative pathway (Scheme 6) which only requires one equivalent of triphenylphosphine per molecule of carbon tetrabromide to give intermediate **13** (Scheme 6) [59]. It has been previously noted that intermediate **2**, while not an active brominating agent in the Appel reaction, is known to assist in the formation of **10** by deprotonating the alcohol to form **11** [57]. However, it is thought that both possible pathways for the Appel reaction are utilised when using solid-supported triphenylphosphine due to the evidence for neighbouring-group interactions (the formation of **1** and **2**), along with site isolation effects ensuring the formation of **13**.

The reactions reported below were therefore performed sequentially using a single monolith. Pleasingly, it was found that after exhausting the monolith of the *gem*-dibromoolefination active species through multiple Ramirez reactions, the monolith could then be used to successfully perform the Appel reaction in flow. Approximately 0.55 mmol of alcohol could be transformed into the corresponding alkyl bromide following approximately 0.80 mmol of successful *gem*-dibromoolefination reactions. When the Appel reaction was performed after the Ramirez reaction, the monolith once again changed colour from dull dark yellow to off-white (depicted in Figure 1, c and d). However, when the loaded monolith was first used for the Appel reaction, there was no conversion observed for a subsequent Ramirez *gem*-dibromoolefination, with only the starting aldehyde being recovered from the output.

These results indicate that the Appel reaction consumes all of the active Ramirez species **2** (Scheme 6), preventing the progress of the Ramirez dibromoolefination. However, if this species is consumed through multiple Ramirez *gem*-dibromoolefination reactions then an alternative brominating agent is utilised to perform the Appel reaction, or alternatively intermediate **2** is not required for the Appel mechanism using intermediate **1**. This is supported by previous observations in the literature that indicate that the predominant pathway for the Appel reaction on solid-support is through intermediates **1** and **2** although overall both pathways are utilised [59]. The possibility of performing the Appel reaction following the use of the same monolith for the Ramirez *gem*-dibromoolefination reaction gives wider synthetic applications for this flow methodology.

## Conclusion

In summary, the monolithic form of triphenylphosphine recently described by our group [37–40] has been successfully applied to the Ramirez *gem*-dibromoolefination reaction in flow. The monolith was loaded with carbon tetrabromide at 0 °C using a single pass protocol to give the active brominating agent. This monolith was then utilised in the Ramirez reaction in flow, transforming a variety of different aldehydes to the corresponding *gem*-dibromoolefins in high yields and excellent purity following only removal of solvent. α-Chiral aldehydes were also successfully transformed, without racemisation of the stereocentre and two ketones bearing electron-withdrawing groups were converted into the desired dibromoolefins in high yield. It was further demonstrated that the same monoliths could be applied to the Appel reaction, giving a small selection of alkyl bromides in high yield and purity without further off-line purification protocols. It was also shown that a single monolith could be used sequentially for Ramirez reactions and then the Appel reaction, but not in reverse order. This indicates that the Appel reaction consumes the Ramirez active brominating agent during the reaction. An alternative mechanistic pathway can ensue if the Appel reaction is performed subsequent to the Ramirez reaction.

## Supporting Information

File 1 Experimental part.

## References

[1] Webb D, Jamison T F (2010). Chem Sci.

[2] Wegner J, Ceylan S, Kirschning A (2012). Adv Synth Catal.

[3] Wenger J, Ceylan S, Kirschning A (2011). Chem Commun.

[4] Hartman R L, McMullen J P, Jensen K F (2011). Angew Chem, Int Ed.

[5] Mason B P, Price K E, Steinbacher J L, Bogdan A R, McQuade D T (2007). Chem Rev.

[6] Ahmed-Omer B, Brandta J C, Wirth T (2007). Org Biomol Chem.

[7] Baxendale I R, Hayward J J, Lanners S, Ley S V, Smith C D, Wirth T (2008). Heterogeneous Reactions. Microreactors in Organic Synthesis and Catalysis.

[8] Myers R M, Roper K A, Baxendale I R, Ley S V, Cossy J, Arseniyadis S (2012). The Evolution of Immobilized Reagents and their Application in Flow Chemistry for the Synthesis of Natural Products and Pharmaceutical Compounds. Modern Tools for the Synthesis of Complex Bioactive Molecules.

[9] García-Verdugo E, Luis S V, Luis S V, Garcia-Verdugo E (2009). Flow Processes Using Polymer-supported Reagents, Scavengers and Catalysts. Chemical Reactions and Process under Flow Conditions.

[10] Baxendale I R, Ley S V, Seeberger P H, Blume T (2007). Solid Supported Reagents in Multi-Step Flow Synthesis. New Avenues to Efficient Chemical Synthesis: Emerging Technologies.

[11] Hodge P (2005). Ind Eng Chem Res.

[12] Kirschning A, Solodenko W, Mennecke K (2006). Chem–Eur J.

[13] Baxendale I R, Deeley J, Griffiths-Jones C M, Ley S V, Saaby S, Tranmer G K (2006). Chem Commun.

[14] Sherrington D C (1998). Chem Commun.

[15] Svec F, Fréchet J M J (1996). Science.

[16] Peters E C, Svec F, Fréchet J M J (1999). Adv Mater.

[17] Svec F, Fréchet J M J (1992). Anal Chem.

[18] Švec F, Fréchet J M J, Švec F, Tennikova T B, Deyl Z (2003). Rigid Macroporous Organic Polymer Monoliths Prepared by Free Radical Polymerization. Monolithic Materials: Preparation, Properties and Applications.

[19] Buchmeiser M R (2007). Polymer.

[20] Švec F, Huber C G (2006). Anal Chem.

[21] Kunz U, Kirschning A, Wen H-L, Solodenko W, Cecilia R, Kappe C O, Turek T (2005). Catal Today.

[22] Jas G, Kirschning A (2003). Chem–Eur J.

[23] Švec F, Tennikova T B, Švec F, Tennikova T B, Deyl Z (2003). Historical Review. Monolithic Materials: Preparation, Properties and Applications.

[24] Nikbin N, Ladlow M, Ley S V (2007). Org Process Res Dev.

[25] Baumann M, Baxendale I R, Ley S V, Nikbin N, Smith C D (2008). Org Biomol Chem.

[26] Baumann M, Baxendale I R, Martin L J, Ley S V (2009). Tetrahedron.

[27] Lange H, Capener M J, Jones A X, Smith C J, Nikbin N, Baxendale I R, Ley S V (2011). Synlett.

[28] Tripp J A, Stein J A, Svec F, Fréchet J M J (2000). Org Lett.

[29] Kirschning A, Altwicker C, Dräger G, Harders J, Hoffmann N, Hoffmann U, Schönfeld H, Solodenko W, Kunz U (2001). Angew Chem, Int Ed.

[30] Kunz U, Schönfeld H, Kirschning A, Solodenko W (2003). J Chromatogr, A.

[31] Solodenko W, Wen H, Leue S, Stuhlmann F, Sourkouni-Argirusi G, Jas G, Schönfeld H, Kunz U, Kirschning A (2004). Eur J Org Chem.

[32] Mennecke K, Kirschning A (2009). Beilstein J Org Chem.

[33] Burguete M I, García-Verdugo E, Vicent M J, Luis S V, Pennemann H, Graf von Keyserling N, Martens J (2002). Org Lett.

[34] Altava B, Burguete M I, García-Verdugo E, Luis S V, Vicent M J (2006). Green Chem.

[35] Bou-Hamdan F R, Krüger K, Tauer K, McQuade D T, Seeberger P H (2013). Aust J Chem.

[36] Sachse A, Galarneau A, Coq B, Fajula F (2011). New J Chem.

[37] Smith C J, Smith C D, Nikbin N, Ley S V, Baxendale I R (2011). Org Biomol Chem.

[38] Smith C J, Nikbin N, Ley S V, Lange H, Baxendale I R (2011). Org Biomol Chem.

[39] Roper K A, Lange H, Polyzos A, Berry M B, Baxendale I R, Ley S V (2011). Beilstein J Org Chem.

[40] (2013). Beilstein TV: "Conducting Appel reactions in flow using a triphenylphosphine monolith".

[41] Desai N B, McKelvie N, Ramirez F (1962). J Am Chem Soc.

[42] Corey E J, Fuchs P L (1972). Tetrahedron Lett.

[43] Uenishi J, Kawahama R, Yonemitsu O, Tsuji J (1996). J Org Chem.

[44] Shen W, Wang L (1999). J Org Chem.

[45] Shen W (2000). Synlett.

[46] Poupon J-C, Boezio A A, Charette A B (2006). Angew Chem, Int Ed.

[47] Fang Y-Q, Lifchits O, Lautens M (2008). Synlett.

[48] Hodge P, Khoshdel E (1985). React Polym.

[49] Bolli M H, Ley S V (1998). J Chem Soc, Perkin Trans 1.

[50] Bernard M, Ford W T (1983). J Org Chem.

[51] Choi M K W, He H S, Toy P H (2003). J Org Chem.

[52] Årstad E, Barrett A G M, Hopkins B T, Köbberling J (2002). Org Lett.

[53] Anilkumar G, Nambu H, Kita Y (2002). Org Process Res Dev.

[54] Cainelli G, Contento M, Manescalchi F, Plessi L (1983). Synthesis.

[55] Sherrington D C, Craig D J, Dagleish J, Domin G, Taylor J, Meehan G V (1977). Eur Polym J.

[56] Harrison C R, Hodge P (1978). J Chem Soc, Chem Commun.

[57] Hodge P, Khoshdel E (1984). J Chem Soc, Perkin Trans 1.

[58] Appel R (1975). Angew Chem, Int Ed Engl.

[59] Tömösközi I, Gruber L, Radics L (1975). Tetrahedron Lett.

[60] Métay E, Hu Q, Negishi E-i (2006). Org Lett.

[61] Sneddon H F, Gaunt M J, Ley S V (2003). Org Lett.

[62] Harding S L (2011). Azadirachtin: towards a second generation synthesis.

