# Supplementary material for: The application of a monolithic triphenylphosphine reagent for conducting Ramirez gem-dibromoolefination reactions in flow
Source: Beilstein J Org Chem. 2013 Sep 2;9:1781–90. doi: 10.3762/bjoc.9.207 (PMC3778394; doi:10.3762/bjoc.9.207)
Supplement: File 1 — Experimental part. [file Beilstein_J_Org_Chem-09-1781-s001.pdf]

Supporting Information

for

The application of a monolithic triphenylphosphine reagent for conducting Ramirez *gem*-dibromoolifination reactions in flow

Kimberley A. Roper[1], Malcolm B. Berry[2] and Steven V. Ley*[1]

Address: [1]Innovative Technology Centre, Department of Chemistry, University of Cambridge, Lensfield Road, Cambridge, Cambridgeshire, CB2 1EW, U.K. and [2]GlaxoSmithKline, Gunnels Wood Road, Stevenage, Hertfordshire, SG1 2NY, U.K.

Email: Steven V. Ley - svl1000@cam.ac.uk

* Corresponding author

Experimental part

List of Contents

General information S2
Formation of the triphenylphosphine monolith S4
Functionalising the monolith with carbon tetrabromide S5
Flow synthesis of *gem*-dibromolefination reactions in flow S6
Flow synthesis of alkyl bromides in flow S14
References S17

General information

Unless otherwise specified, reagents were obtained from commercial sources and used without further purification. Divinylbenzene (80% technical grade) was obtained from Sigma-Aldrich, and diphenyl(4-vinylphenyl)phosphine (90% purity) was obtained from Hokko Chemical Industry Co., Ltd. 3-Phenylpropiolaldehyde was distilled before use (118 °C, 13 mmHg).

Solvents: Anhydrous CH_2Cl_2 (technical grade) was passed through an activated alumina column followed by a catalytic copper purification column under an atmosphere of dry argon immediately before use [1].

Chromatography: Analytical thin-layer chromatography was performed on precoated glass-backed plates (Merck Kieselgel 60 F_{254}) and visualised by ultraviolet irradiation (254 nm) Retention factors are reported as R_f.

Atom labelling: Atom labels are according to the numbering applied to the compound figure, originating at the functional group that has been introduced (usually the bromine). The compounds are named according to the guidelines specified by the International Union of Pure and Applied Chemistry (IUPAC). As a result the numbering system in the compound name does not correspond to the atom labels.

Melting points (mp): Determined using an OptiMelt melting point system available from Stanford Research Systems calibrated against vanillin (83 °C), phenacetin (136 °C) and caffeine (237 °C). For recrystallised samples, the solvent system is listed in parenthesises after the melting point range as are any recrystallisation solvent systems reported in the literature.

Liquid chromatography-mass spectrometry (LCMS): Performed on an Agilent HP 1100 series chromatograph (Mercury Luna 3µ C18 (2) column) attached to a Waters ZQ2000 mass spectrometer with ESCi ionisation source in electrospray ionisation (ESI) mode. Elution was carried out at a flow rate of 0.60 mL/min using a reverse phase gradient of MeCN–H_2O containing 0.1% formic acid. Gradient = 0–1 min: hold MeCN 5%, 1–4 min: ramp MeCN 5–95%, 4–5 min: hold MeCN 95%, 5–7 min: ramp MeCN 95–5%, 7–8 min: hold MeCN 5%. Retention time is reported as t_R.

Optical rotations: Measured on a PerkinElmer Model 343 digital polarimeter using a sodium lamp (589 nm) as the light source over a path length of 10 cm. $[\alpha]_D$ values are reported in units of 10^{-1}deg cm^{2}g^{-1} at concentration (*c*) in g per 100 mL.

NMR spectroscopy: ^{1}H NMR spectra were recorded on a Bruker DPX-600 (600 MHz) spectrometer with the deuterated solvent as an internal deuterium lock. Chemical shift data (δ) are given in parts per million relative to tetramethylsilane (external standard), and calibrated using the residual monoprotic solvent (7.26 ppm $CHCl_3$) [2] as an internal reference. The centre of each signal is reported with the exception of multiplets, for which the range of ppm values covered by the signal is reported. The multiplicity of a signal is indicated as: s – singlet, d – doublet, t – triplet, m – multiplet, br – broad, app – apparent or combinations of these coupling constants (*J*) are recorded to the nearest 0.1 Hz.
^{13}C NMR spectra were recorded on a Bruker DRX-600 (150 MHz) spectrometer with broadband proton decoupling with the deuterated solvent as internal deuterium lock.

Chemical shift data (δ) are given in parts per million relative to tetramethylsilane (external standard), and calibrated using the deuterated solvent (77.16 ppm $CDCl_3$) [2] as an internal reference.
DEPT 135, COSY, HMQC, HMBC spectra and nOe experiments were used to aid in the assignment of signals in ^{1}H and ^{13}C NMR spectra where appropriate.

IR Spectroscopy: Spectra were recorded on a PerkinElmer Spectrum One FTIR spectrometer using universal Attenuated Total Reflectance (ATR) sampling accessories as a thin film. Letters in parentheses refer to relative absorbance with respect to the most intense peak: w – weak (<40%), m – medium (40–75%), s – strong (>75%).

Elemental composition microanalysis: Performed by the Microanalytical Laboratories at the Department of Chemistry, University of Cambridge and results are reported to one decimal place. Limit: ±0.5% that of calculated.

High resolution mass spectrometry (HRMS): Performed on a Bruker BioApex II 4.7e FTICR utilising either positive electrospray ionisation (ESI^+) or positive electron ionisation (EI^+) source equipped with a direct insertion probe. The mass reported is that containing the most abundant isotope (^{79}Br). Limit: ±5 parts per million.

X-ray crystallography: Recorded by Dr. John Davies at the Department of Chemistry, University of Cambridge using a Nonius Kappa CCD detector. CIF-file numbers, space groups and cell parameters are listed as part of the compound characterisation. Crystal structure images presented in this thesis were produced using ORTEP-3 for Windows [3].

Flow equipment: Flow experiments used anhydrous solvents kept under an atmosphere of argon in oven-dried glassware (200 °C). Polymeric reagents were contained in glass Omnifit® columns with monolithic reagents formed and used in glass Omnifit® columns, sealed for polymerisation with custom-made PTFE end pieces and heated using a Vapourtec R4 system. Monoliths and columns were otherwise connected to a Uniqsis FlowSyn flow system with standard Omnifit® column end pieces, tubing connectors and PTFE tubing. Where possible the flow direction through the monoliths was against gravity (input at the bottom of the monolith, output at the top) to negate any gravitational effects.

Formation of the triphenylphosphine monolith

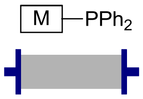

The preparation scale specified below forms a monolith that fills a 7.85 mL glass Omnifit® column (10 mm i.d. × 100 mm length). Multiple monoliths can be made from the same stock solution by multiplying the values below as appropriate.

A stirred solution of diphenyl(4-vinylphenyl)phosphine (1.67 g, 5.8 mmol), divinylbenzene (1.01 g, 7.7 mmol), styrene (0.23 g, 2.2 mmol) and 1-dodecanol (2.25 g, 12.1 mmol) was heated to 50 °C. Upon complete dissolution, dibenzoyl peroxide (35.0 mg, 0.14 mmol, 0.9 mol % with respect to the monomer and crosslinker) was added and the mixture stirred for a further 5 min at 50 °C. A 10 mm i.d. × 100 mm length glass column was sealed at one end with a custom-made PTFE end plug, filled to 70 mm height with the aforementioned solution, and sealed with another PTFE end plug. The column was heated at 92 ºC using a Vapourtec R4 convection heater unit and after 1 h the clear solution became white and cloudy. The monolith was then heated at 92 °C for another 47 h, resulting in a rigid white monolith. Following polymerisation, the monolith was allowed to cool to room temperature, and the end seals exchanged for fritted tubing connectors. The porogen was then removed from the monolith. The following procedure was found to remove the vast majority of dodecanol. The monolith was heated to 60 ºC and CH_2Cl_2 was passed through the monolith at 0.20 mL/min without a back pressure regulator for 1 h. The monolith was then inverted, the tubing input and outputs connected to the monolith swapped and the process repeated. A 100 psi back pressure regulator was added to the end of the system and further CH_2Cl_2 passed through the monolith at 0.20 mL/min for an additional hour. The flow rate was then gradually increased to 1.50 mL/min over the next hour. The monolith was once again inverted, the input and output swapped and the previous two steps repeated. CH_2Cl_2 was passed through the monolith at 0.20 mL/min while it was cooled to room temperature and then the flow rate incrementally increased to 1.50 mL/min over the next hour. The monolith was inverted, input and output swapped and the previous step repeated.

Elemental analysis found C 86.89, H 6.79, P 5.74% (loading = 1.85 mmol P per g monolith); Dry weight = 2.5 g; IR (thin film) ν_{max}/cm^{-1} 2923.8 (w), 2333.6 (w), 1952.9 (w), 1585.2 (w), 1479.3 (w), 1434.1 (w), 1403.5 (w), 1181.9 (w), 1092.6 (w), 1016.6 (w), 989.6 (w), 902.3 (w), 825.0 (w), 797.2 (w), 742.0 (m), 695.1 (s).

Functionalising the monolith with carbon tetrabromide

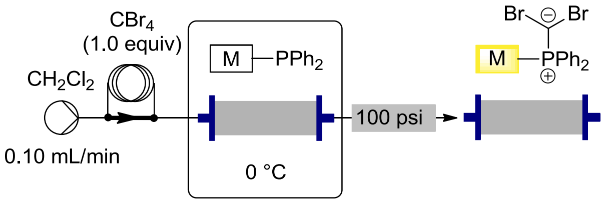

A stream of CH_2Cl_2 was passed through the triphenylphosphine monolith, and then through a 100 psi back pressure regulator at 0.10 mL/min for 20 min while the monolith was cooled to 0 °C by submersion in an ice-water bath. A solution of carbon tetrabromide (1.82 g, 5.5 mmol) in CH_2Cl_2 (10 mL) was prepared and 4 mL of this solution was loaded into a sample loop. The sample loop was then switched inline and the carbon tetrabromide solution passed through the monolith at 0.10 mL/min. The output stream was collected for 1 h. The rest of the carbon tetrabromide solution was passed through the monolith via this method in two further injections. The monolith became bright yellow during the loading process, and an accompanying overall rise in pressure of approximately 2 bar was observed. CH_2Cl_2 was then passed through the monolith at 0.50 mL/min for 30 mins at 0 °C. The solvent was removed in vacuo and approximately 1.40 g carbon tetrabromide was isolated, indicating that 0.42 g (1.3 mmol) had reacted during the loading process. The monolith could be sealed at both ends and stored for up to 7 days at −20 °C in CH_2Cl_2 between experiments.

A sample of this monolith was collected under an inert atmosphere and submitted for analysis.
Elemental analysis found C 71.79, H 5.69, P 4.54, Br 15.31% (number of equivalents of Br to P = 1.31:1); IR (thin film) ν_{max}/cm^{-1} 2925.4 (w), 2347.8 (w), 1598.1 (w), 1484.7 (w), 1436.7 (w), 1406.3 (w), 1187.2 (w), 1157.5 (w), 1119.5 (m), 1064.3 (w), 1014.8 (w), 996.7 (w), 905.8 (w), 827.0 (w), 797.9 (w), 746.2 (m), 723.2 (m), 694.2 (s).

Flow synthesis of *gem*-dibromolefination reactions in flow

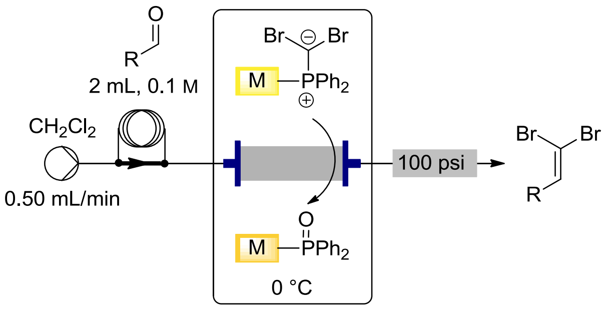

A stream of CH_2Cl_2 was passed through the previously functionalised monolith, cooled to 0 °C, and then through a 100 psi back pressure regulator at 0.50 mL/min. A solution of the aldehyde or ketone (0.2 mmol) in CH_2Cl_2 (2 mL) was prepared and injected into a 2 mL sample loop. The sample loop was switched inline and the output stream was collected for 1 h 15 min. The monolith changed colour from bright yellow to a dull dark yellow colour. The solvent was then removed in vacuo to yield the product. Each monolith was found to have an average approximate active loading of 0.80 mmol, calculated through experimental results.

Analysis of the dull dark yellow region gave the following data (sample collected under an inert atmosphere):
Elemental analysis found C 75.00, H 5.95, P 5.01, Br 13.35% (number of equivalents of Br to P = 1.03:1); IR (thin film) ν_{max}/cm^{-1} 2912.7 (w), 1595.6 (w), 1486.6 (w), 1436.8 (m), 1403.0 (w), 1310.1 (w), 1120.3 (m), 1066.3 (w), 1014.8 (w), 996.9 (w), 989.0 (w), 903.7 (w), 827.4 (m), 796.7 (w), 747.1 (m), 721.4 (m), 693.7 (s).

All yields reported below were obtained *via* the above procedure unless otherwise specified.

1-(2,2-Dibromovinyl)-2-methoxybenzene (Table 1, entry 1) [4]

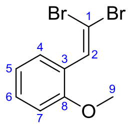

Isolated as a dark yellow solid (46.5 mg, 80%); LCMS t_R 5.22 min, UV-trace only; mp 31–32 °C, [lit. [4] 31–33 °C]; ^{1}H NMR (600 MHz, $CDCl_3$) δ_H: 7.70 (d, *J* = 7.6 Hz, 1H, H^7), 7.61 (s, 1H, H^2), 7.33 (app t, *J* = 7.8 Hz, 1H, H^6), 6.97 (app t, *J* = 7.5 Hz, 1H, H^5), 6.88 (d, *J* = 8.3 Hz, 1H, H^4), 3.84 (s, 3H, H^9); ^{13}C NMR (150 MHz, $CDCl_3$) δ_C: 156.7 (*C*, C^8), 133.1 (*C*H, C^2), 130.1 (*C*H, C^6), 129.3 (*C*H, C^7), 124.5 (*C*, C^3), 120.3 (*C*H, C^5) 110.6 (*C*H, C^4) 89.9 (CBr_2, C^1), 55.6 (CH_3, C^9); IR (thin film) ν_{max}/cm^{-1} 3675.5 (w), 2963.0 (w, br), 2836.1 (w), 1597.7 (w), 1579.2 (w), 1484.0 (m), 1461.6 (m), 1434.8 (m), 1312.4 (w), 1289.8 (w), 1243.8 (s), 1193.6 (w), 1175.4 (w), 1162.4 (w), 1109.8 (m), 1049.7 (m), 1026.2 (m), 936.4 (w), 875.0 (m), 843.8 (m), 808.1 (s), 747.5 (s), 728.8 (m); HRMS *m/z* (EI^+) found 289.8937 ($[M]^+$, $C_9H_8OBr_2$ requires 289.8936), Δ = 0.3 ppm. The data for this compound is in agreement with that reported [4].

4-(2,2-Dibromovinyl)benzonitrile (Table 1, entry 2) [5]

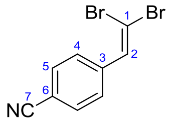

Isolated as a pale yellow solid (54.5 mg, 95%); mp 90–91 °C, [lit. [5] 79–80 °C]; LCMS t_R 5.02 min, UV-trace only; ^{1}H NMR (600 MHz, $CDCl_3$) δ_H: 7.66 (d, *J* = 8.5 Hz, 2H, H^4), 7.63 (d, *J* = 8.4 Hz, 2H, H^5), 7.50 (s, 1H, H^2); ^{13}C NMR (150 MHz, $CDCl_3$) δ_C: 139.8 (*C*, C^6), 135.3 (*C*H, C^2), 132.4 (2 × *C*H, C^5), 129.1 (2 × *C*H, C^4), 118.6 (*C*N, C^7), 112.1 (*C*, C^3), 93.6 (CBr_2, C^1); IR (thin film) ν_{max}/cm^{-1} 2924.0 (w), 2226.8 (w), 1599.7 (m), 1552.8 (w), 1497.6 (w), 1407.3 (m), 1297.8 (w), 1261.2 (w), 1176.7 (w), 1110.1 (w), 1018.0 (w), 971.1 (w), 956.1 (w), 874.6 (s), 848.4 (s), 814.9 (s), 804.7 (m), 790.1 (m), 719.0 (m), 698.0 (m); Elemental analysis found C 37.81, H 1.67, N 4.85, Br 55.38% ($C_9H_5NBr_2$ requires C 37.67, H 1.76, N 4.88, Br 55.69%); HRMS *m/z* (EI^+) found 284.8787 ($[M]^+$, $C_9H_5NBr_2$ requires 284.8783), Δ = 1.3 ppm. The data for this compound is in agreement with that reported [5].

1-(2,2-Dibromovinyl)-2-nitrobenzene (Table 1, entry 3) [6]

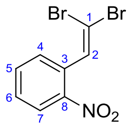

Isolated as a pale green solid (57.0 mg, 93%); mp 56–57 °C, [lit. [6] 61–62 °C]; LCMS t_R 5.00 min, *m/z* (ESI^+) 305 ($[M + H]^+$); ^{1}H NMR (600 MHz, $CDCl_3$) δ_H: 8.12 (d, *J* = 8.1 Hz, 1H, H^4), 7.78 (s, 1H, H^2), 7.67 (app t, *J* = 7.2 Hz, 1H, H^5), 7.60 (d, *J* = 7.4 Hz, 1H, H^7), 7.54 (app t, *J* = 7.5 Hz, 1H, H^6); ^{13}C NMR (150 MHz, $CDCl_3$) δ_C: 146.9 (CNO_2, C^8), 134.2 (*C*H, C^2), 133.7 (*C*H, C^6), 131.8 (*C*H, C^7), 131.5 (*C*, C^3), 129.6 (*C*H, C^5), 125.0 (*C*H, C^4), 93.4 (CBr_2, C^1); IR (thin film) ν_{max}/cm^{-1} 3099.2 (w), 3083.3 (w), 3047.2 (w), 2847.8 (w), 1608.9

(w), 1570.6 (w), 1510.2 (m), 1473.0 (w), 1439.9 (w), 1391.4 (w), 1342.0 (s), 1308.1 (m), 1269.6 (w), 1202.3 (w), 1142.6 (w), 1077.1 (w), 1043.8 (w), 996.1 (w), 960.4 (w), 887.7 (m), 861.4 (m), 837.0 (m), 787.4 (s), 737.9 (s), 694.1 (s), 666.4 (m); Elemental analysis found C 31.49, H 1.63, N 4.44, Br 51.73% ($C_8H_5NO_2Br_2$ requires C 31.30, H 1.64, N 4.56, Br 52.07%); HRMS *m/z* (EI^+) found 225.9501 ($[M - Br]^+$, $C_8H_5NO_2Br$ requires 225.9498), Δ = 1.2 ppm. The data for this compound is in agreement with that reported [6].

4-(2,2-Dibromovinyl)-1,1'-biphenyl (Table 1, entry 4) [7]

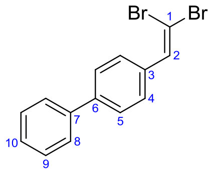

Isolated as a white crystalline solid (66.3 mg, 98%); mp 104–106 °C, [lit. [8] 105–106 °C (hexane)]; LCMS t_R 4.62 min, UV-trace only; ^{1}H NMR (600 MHz, $CDCl_3$) δ_H: 7.65–7.60 (m, 6H, H^4, H^5 and H^8), 8.57 (s, 1H, H^2), 7.46 (app t, J = 7.4 Hz, 2H, H^9), 7.38 (t, J = 7.2 Hz, 1H, H^{10}); ^{13}C NMR (150 MHz, $CDCl_3$) δ_C: 141.4 (*C*, C^7), 140.5 (*C*, C^6), 136.6 (*C*H, C^2), 134.33 (*C*, C^3), 129.0 (2 × *C*H, C^9), 129.0 (2 × *C*H, C^4), 127.8 (*C*H, C^{10}), 127.2 (2 × *C*H, C^8), 127.2 (2 × *C*H, C^5), 89.6 (*C*Br_2, C^1); IR (thin film) ν_{max}/cm^{-1} 3031.7 (w), 3008.7 (w), 2924.2 (w), 1752.2 (w), 1676.9 (w), 1589.6 (w), 1552.3 (w), 1481.9 (w), 1448.9 (w), 1406.8 (w), 1330.0 (w), 1299.8 (w), 1263.0 (w), 1195.2 (w), 1181.9 (w), 1161.3 (w), 1134.9 (w), 1075.2 (w), 1040.5 (w), 1003.0 (w), 949.3 (w), 913.7 (w), 876.4 (m), 863.6 (m), 848.2 (w), 826.0 (m), 786.3 (m), 759.5 (s), 717.5 (m), 706.9 (w), 686.6 (s); HRMS *m/z* (EI^+) found 335.9146 ($[M]^+$, $C_{14}H_{10}Br_2$ requires 335.9144), Δ = 0.7 ppm. The data for this compound is in agreement with that reported [7].

(5,5-Dibromopent-4-en-2-yl)benzene (Table 1, entry 5)

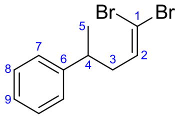

Isolated as a colourless oil (48.2 mg, 79%); LCMS R_t 5.48 min, *m/z* (ESI^+) 145 ($[M - Br_2 + H]^+$); ^{1}H NMR (600 MHz, $CDCl_3$) δ_H: 7.32 (app t, J = 7.6 Hz, 2H, H^8), 7.24–7.19 (m, 3H, H^7 and H^9), 6.31 (t, J = 7.2 Hz, 1H, H^2), 2.89 (app sextet, J = 7.1 Hz, 1H, H^4), 2.39 (app t, J = 7.3 Hz, 2H, H^3), 1.30 (d, J = 7.0 Hz, 3H, H^5); ^{13}C NMR (150 MHz, $CDCl_3$) δ_C: 145.9 (*C*, C^6), 137.3 (*C*H, C^2), 128.7 (2 × *C*H, C^8), 127.0 (2 × *C*H, C^7), 126.6 (*C*H, C^9), 89.6 (*C*Br_2, C^1), 41.6 (*C*H_2, C^3), 38.9 (*C*H, C^4), 21.7 (*C*H_3, C^5); IR (thin film) ν_{max}/cm^{-1} 3027.6 (w), 2962.7 (w), 2928.6 (w), 1736.0 (w), 1621.4 (w), 1602.8 (w), 1493.9 (w), 1452.0 (w), 1377.5 (w), 1326.0 (w), 1278.4 (w), 1246.7 (w), 1191.6 (w), 1159.5 (w), 1086.8 (w), 1027.8 (w), 907.6 (w), 889.2 (w), 828.4 (w), 784.7 (m), 759.1 (m), 736.5 (w), 698.2 (s); HRMS *m/z* (EI^+) found 223.0121 ($[M - Br]^+$, $C_{11}H_{12}Br$ requires 223.0117), Δ = 1.7 ppm.

(2,2-Dibromovinyl)cyclopropane (Table 1, entry 6) [9]

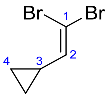

Isolated as a colourless oil (35.2 mg, 78%); LCMS t_R 5.04 min, *m/z* (ESI$^+$) 149 ([M – Br + H]$^+$); ^{1}H NMR (600 MHz, $CDCl_3$) δ_H: 5.78 (d, *J* = 9.3 Hz, 1H, H^2), 1.67–1.61 (m, 1H, H^3), 0.89–0.85 (m, 2H, H^4), 0.55–0.52 (m, 2H, H^4); ^{13}C NMR (150 MHz, $CDCl_3$) δ_C: 142.3 (*C*H, C^2), 85.2 (*C*Br_2, C^1), 15.4 (C*H*, C^3), 6.8 (2 × *C*H_2, C^4); IR (thin film) ν_{max}/cm^{-1} 3086.6 (w), 3009.1 (w), 1455.9 (w), 1431.5 (w), 1355.9 (w), 1259.0 (w), 1192.5 (w), 1172.6 (w), 1092.9 (w), 1050.7 (w), 1023.6 (w), 953.0 (s), 931.5 (w), 881.2 (m), 831.0 (w), 810.7 (w), 780.4 (s), 764.2 (s); HRMS *m/z* (EI$^+$) found 233.8837 ([M]$^+$, $C_5H_6Br_2$ requires 223.8831), Δ = 2.9 ppm. The data for this compound is in agreement with that reported [9].

1,1-Dibromo-4-phenyl-1-buten-3-yne (Table 1, entry 7) [10]

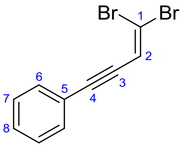

Isolated as a yellow oil (47.7 mg, 83%); LCMS t_R 5.41 min, UV-trace only; ^{1}H NMR (600 MHz, $CDCl_3$) δ_H: 7.49 (dd, *J* = 8.2, 1.5 Hz, 2H, H^6), 7.38–7.32 (m, 3H, H^8 and H^7), 6.78 (s, 1H, H^2); ^{13}C NMR (150 MHz, $CDCl_3$) δ_C: 131.7 (2 × *C*H, C^6), 129.2 (*C*H, C^8), 128.6 (2 × *C*H, C^7), 122.6 (*C*, C^5), 119.8 (*C*H, C^2), 101.9 (*C*Br_2, C^1), 97.3 (Ph*C*C, C^4), 86.3 (PhC*C*, C^3); IR (thin film) ν_{max}/cm^{-1} 3017.9 (w), 2200.1 (w), 1597.8 (w), 1570.6 (w), 1488.3 (m), 1441.9 (w), 1275.4 (w), 1255.0 (w), 1176.2 (w), 1157.4 (w), 1068.8 (w), 1034.6 (w), 1014.6 (w), 996.9 (w), 914.3 (w), 843.9 (s), 820.5 (m), 752.5 (s), 717.7 (w), 686.7 (s); HRMS *m/z* (EI$^+$) found 283.8825 ([M]$^+$, $C_{10}H_6Br_2$ requires 283.8831), Δ = 2.2 ppm. The data for this compound is in agreement with that reported [10].

2-(2,2-Dibromovinyl)furan (Table 1, entry 8) [11]

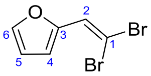

Isolated as a pale yellow oil (48.9 mg, 97%); LCMS t_R 5.05 min, *m/z* (ESI$^-$) 251 ([M − H]$^-$); ^{1}H NMR (600 MHz, $CDCl_3$) δ_H: 7.44 (s, 1H, H^6), 7.41 (s, 1H, H^2), 6.95 (d, *J* = 2.6 Hz, 1H, H^4), 6.46 (br s, 1H, H^5); ^{13}C NMR (150 MHz, $CDCl_3$) δ_C: 150.2 (*C*, C^3), 142.7 (*C*H, C^6), 126.6 (*C*H, C^2), 111.7 (*C*H, C^5), 111.6 (*C*H, C^4), 87.3 (*C*Br_2, C^1); IR (thin film) ν_{max}/cm^{-1} 3031.6 (w), 1797.7 (w),1605.0 (w), 1560.3 (w), 1481.0 (m), 1379.3 (w), 1285.6 (w), 1258.1 (w), 1237.9 (w), 1211.3 (w), 1175.2 (w), 1141.7 (w), 1086.0 (w), 1019.2 (m), 946.3 (m), 930.6 (w), 885.4 (w), 834.7 (s), 807.7 (w), 737.1 (s), 697.8 (s), 666.7 (s); HRMS *m/z* (EI$^+$) found 249.8633 ([M]$^+$, $C_6H_4OBr_2$ requires 249.8623), Δ = 3.8 ppm. The data for this compound is in agreement with that reported [11].

(*E*)-2-(4,4-Dibromobuta-1,3-dien-1-yl)furan (Table 1, entry 9) [10]

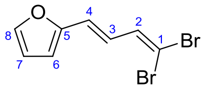

Isolated as a pale green solid (50.4 mg, 91%); mp 32–35 °C; LCMS t_R 5.28 min, *m/z* (ESI$^+$) 279 ([M + H]$^+$); ^{1}H NMR (600 MHz, $CDCl_3$) δ_H: 7.43 (s, 1H, H^8), 7.02 (d, *J* = 10.6 Hz, 1H, H^2), 6.68 (dd, *J* = 15.5, 10.6 Hz, 1H, H^3), 6.49 (d, *J* = 15.5 Hz, 1H, H^4), 6.42–6.40 (m, 2H, H^6 and H^7); ^{13}C NMR (150 MHz, $CDCl_3$) δ_C: 152.5 (*C*, C^5), 143.3 (*C*H, C^8), 136.8 (*C*H, C^2), 123.7 (*C*H, C^3), 122.7 (*C*H, C^4), 112.2 (*C*H, C^7), 110.9 (*C*H, C^6), 91.5 (*C*Br_2, C^1); IR (thin film) ν_{max}/cm^{-1} 3142.7 (w), 3118.7 (w), 3043.1 (w), 3009.7 (w), 2087.6 (w), 1831.1 (w), 1797.5 (w), 1744.8 (w), 1679.7 (w), 1615.4 (w), 1533.0 (w), 1502.0 (w), 1471.6 (w), 1385.9 (w), 1298.6 (w), 1260.8 (w), 1236.2 (w), 1201.8 (w), 1152.4 (w), 1072.7 (w), 1014.7 (m), 952.8 (s), 928.1 (m), 883.6 (w), 874.8 (m), 813.9 (s), 760.6 (m), 735.7 (s), 665.8 (w); HRMS *m/z* (EI$^+$) found 275.8791 ([M]$^+$, $C_8H_6OBr_2$ requires 275.8780), Δ = 4.0 ppm. The data for this compound is in agreement with that reported [10].

3-(2,2-Dibromovinyl)thiophene (Table 1, entry 10) [12]

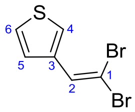

Isolated as a yellow oil (46.8 mg, 87%); LCMS t_R 5.17 min, *m/z* (ESI$^+$) 269 ([M + H]$^+$); ^{1}H NMR (600 MHz, $CDCl_3$) δ_H: 7.69–7.68 (m, 1H, H^4), 7.48 (s, 1H, H^2), 7.38 (dd, *J* = 5.0, 0.9 Hz, 1H, H^6), 7.31 (dd, *J* = 5.0, 3.0 Hz, 1H, H^5); ^{13}C NMR (150 MHz, $CDCl_3$) δ_C: 136.2 (*C*, C^3), 131.6 (*C*H, C^2), 127.7 (*C*H, C^6), 125.7 (*C*H, C^4), 125.4 (*C*H, C^5), 88.6 (*C*Br_2, C^1); IR (thin film) ν_{max}/cm^{-1} 3098.8 (w), 3017.1 (w), 2921.1 (w), 2849.2 (w), 1591.4 (w), 1567.8 (w), 1522.5 (w), 1500.4 (w), 1457.0 (w), 1413.0 (w), 1400.8 (w), 1363.1 (w), 1287.6 (w), 1242.2 (w), 1204.2 (w), 1158.9 (w), 1081.6 (w), 957.7 (m), 939.3 (w), 882.9 (m), 854.2 (m), 834.9 (m), 822.7 (m), 807.2 (s), 768.5 (s), 687.3 (m), 674.5 (m); HRMS *m/z* (EI$^+$) found 265.8382 ([M]$^+$, $C_6H_4SBr_2$ requires 265.8395), Δ = 4.9 ppm. The data for this compound is in agreement with that reported [12].

3-(2,2-Dibromovinyl)pyridin-1-ium bromide (Table 1, entry 11) [13]

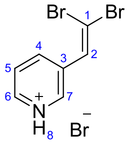

Output collected for 2 h. Isolated a white crystalline solid (28.5 mg, 41%); mp 54–56 °C, [lit. [13] 57–59 °C]; LCMS t_R 2.71 min, *m/z* (ESI$^+$) 264 ([M − Br]$^+$); ^{1}H NMR (600 MHz, $CDCl_3$) δ_H: 8.71 (br s, 1H, H^7), 8.58 (br s, 1H, H^6), 8.00 (d, *J* = 7.9 Hz, 1H, H^4), 7.47 (s, 1H, H^2), 7.35 (br s, 1H, H^5), 3.72 (br s, 1H, H^8);^{13}C NMR (150 MHz, $CDCl_3$) δ_C: 149.3 (*C*H, C^7), 148.9 (*C*H, C^6), 135.6 (*C*H, C^4), 133.5 (*C*H, C^2), 131.9 (*C*, C^3), 123.6 (*C*H, C^5), 92.8 (*C*Br_2, C^1); IR (thin film) ν_{max}/cm^{-1} 3010.7 (w), 2571.8 (w, br), 2059.0 (w), 1883.2 (w), 1601.5 (w), 1579.4 (w), 1567.7 (w), 1546.0 (w), 1479.5 (w), 1431.1 (w), 1408.1 (m), 1375.6 (w), 1354.6

(w), 1338.9 (w), 1322.0 (w), 1279.8 (w), 1260.6 (w), 1246.1 (w), 1222.3 (w), 1177.9 (w), 1125.4 (w), 1094.5 (w), 1044.3 (w), 1023.1 (m), 983.1 (w), 956.0 (w), 930.4 (w), 892.9 (w), 871.6 (s), 842.9 (s), 801.6 (m), 780.0 (s), 731.3 (w), 703.3 (s), 675.0 (m); HRMS *m/z* (EI^+) found 260.8773 ($[M – H − Br]^+$, $C_7H_5NBr_2$ requires 260.8783), Δ = 3.8 ppm; HRMS *m/z* (ESI^+) found 261.8850 ($[M − Br]^+$, $C_7H_6NBr_2$ requires 261.8861), Δ = 4.5 ppm; X-ray crystallography: File reference SL1218; Formula: $C_7H_6NBr_3$; Unit cell parameters; a 6.5917(2) b 19.1978(7) c 8.0408(3) α 90.00 β 104.636(2) γ 90.00 space group P2(1)/c. The data for this compound is in agreement with that reported [13].

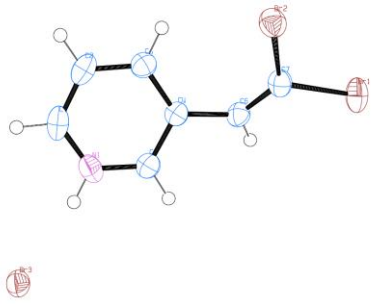

(2*R*,3*R*,5*S*)-5-(2,2-Dibromovinyl)-2,3-dimethoxy-2,3-dimethyl-1,4-dioxane (Table 2, entry 1) [14]

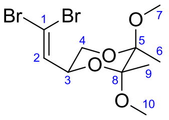

Isolated as a pale yellow oil that crystallized upon storing at –20 °C to afford pale yellow crystals (68.1 mg, 95%); mp 41–45 °C; $[\alpha]_D^{27.4}$ = –145.7 (*c* = 1.05, $CHCl_3$), [lit [14] $[\alpha]_D^{25}$ = –77.6 (*c* = 1.05, $CHCl_3$)]; LCMS t_R 4.92 min, UV-trace only; ^{1}H NMR (600 MHz, $CDCl_3$) δ_H : 6.41 (d, *J* = 8.1 Hz, 1H, H^2), 4.67–4.63 (m, 1H, H^3), 3.61 (app t, *J* = 11.3 Hz, 1H, $H_{ax}{}^4$), 3.49 (dd, *J* = 11.0, 2.2 Hz, 1H, $H_{eq}{}^4$), 3.33 (s, 3H, H^7), 3.27 (s, 3H, H^{10}), 1.29 (s, 6H, H^6 and H^9); ^{13}C NMR (150 MHz, $CDCl_3$) δ_C : 134.7 (*C*H, C^2), 99.4 (*C*, C^8), 98.0 (*C*, C^5), 94.1 (*C*Br_2, C^1), 69.1 (*C*H, C^3), 60.3 (*C*H_2, C^4), 48.5 (O*C*H_3, C^7), 48.3 (O*C*H_3, C^{10}), 17.9 (*C*H_3, C^6 or C^9), 17.7 (*C*H_3, C^6 or C^9); IR (thin film) ν_{max}/cm^{-1} 3030.3 (w), 2988.9 (w), 2964.6 (w), 2946.8 (w), 2924.2 (w), 2834.4 (w), 1629.1 (w), 1582.5 (w), 1456.2 (w), 1438.7 (w), 1371.6 (m), 1351.5 (w), 1299.5 (w), 1232.0 (w), 1213.4 (w), 1167.7 (w), 1140.1 (m), 1111.2 (s), 1079.0 (s), 1051.5 (m), 1031.7 (s), 946.5 (m), 878.5 (s), 850.9 (m), 795.9 (s), 725.6 (w), 662.0 (m); HRMS *m/z* (EI^+) found 326.9218 ($[M − OCH_3]^+$, $C_9H_{13}O_3Br_2$ requires 326.9226), Δ = 2.4 ppm.

(S)-4-((S)-4,4-Dibromo-2-methylbut-3-en-1-yl)-2,2-dimethyl-1,3-dioxolane (Table 2, entry 2)

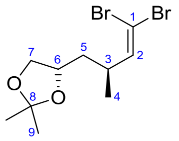

The starting aldehyde was supplied as a 9.4:1 mixture of diasteroisomers (determined by ^{1}H NMR[1]).

Isolated as a pale pink oil (54.8 mg, 84%); determined to be in a 9.5:1 ratio of diastereoisomers by ^{1}H NMR[2]; $[\alpha]_D^{27.4}$ = +7.0 (c = 1.00, $CHCl_3$); LCMS t_R 5.20 min, UV-trace only; Major diastereoisomer: ^{1}H NMR (600 MHz, $CDCl_3$) δ_H: 6.21 (d, J = 9.5 Hz, 1H, H^2), 4.10–4.05 (m, 2H, H^6 and H^7), 3.54 (dt, J = 6.3, 0.7 Hz, 1H, H^7), 2.57–2.50 (m, 1H, H^3), 1.76–1.71 (m, 1H, H^5), 1.51–1.47 (m, 1H, H^5), 1.40 (s, 3H, H^9), 1.35 (s, 3H, H^9), 1.06 (d, J = 6.7 Hz, 3H, H^4); ^{13}C NMR (150 MHz, $CDCl_3$) δ_C: 143.4 (CH, C^2), 109.0 ($C(CH_3)_2$, C^8), 88.2 (CBr$_2$, C^1), 73.9 (OCH, C^6), 69.8 (OCH$_2$, C^7), 39.9 (CH$_2$, C^5), 35.7 (CHCH$_3$, C^3), 27.2 (C(CH$_3$)$_2$, C^9), 25.9 (C(CH$_3$)$_2$, C^9), 19.5 (CH$_3$, C^4); Minor diastereoisomer: ^{1}H NMR (600 MHz, $CDCl_3$) δ_H: 6.19 (d, J = 9.6 Hz, 1H, H^2), 4.05–4.01 (m, 2H, H^6 and H^7), 3.45 (app t, J = 7.5 Hz, 1H, H^7), 2.70–2.63 (m, 1H, H^3), 1.72–1.67 (m, 1H, H^5), 1.54–1.48 (m, 1H, H^5), 1.40 (s, 3H, H^9), 1.35 (s, 3H, H^9), 1.06 (d, J = 6.6 Hz, 3H, H^4); ^{13}C NMR (150 MHz, $CDCl_3$) δ_C: 143.5 (CH, C^2), 108.8 ($C(CH_3)_2$, C^8), 88.2 (CBr$_2$, C^1), 74.4 (OCH, C^6), 69.7 (OCH$_2$, C^7), 39.9 (CH$_2$, C^5), 36.1 (CHCH$_3$, C^3), 27.1 (C(CH$_3$)$_2$, C^9), 25.9 (C(CH$_3$)$_2$, C^9), 19.8 (CH$_3$, C^4); IR (thin film) ν_{max}/cm^{-1} 2983.5 (w), 2961.9 (w), 2929.3 (w), 2871.7 (w), 1616.0 (w), 1455.4 (w), 1378.2 (m), 1369.3 (m), 1247.7 (m), 1214.4 (m), 1160.2 (m), 1151.5 (w), 1081.0 (m), 1055.9 (s), 1004.4 (w), 961.2 (w), 950.3 (w), 915.6 (w), 883.8 (w), 856.3 (m), 823.6 (m), 782.2 (s), 670.7 (w); HRMS m/z (EI$^+$) found 325.9511 ([M]$^+$, $C_{10}H_{16}O_2Br_2$ requires 325.95116), Δ = 3.1 ppm.

(S)-(((4,4-Dibromo-2-methylbut-3-en-1-yl)oxy)methyl)benzene (Table 2, entry 3) [15]

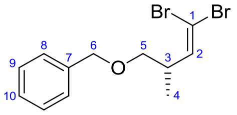

Isolated as a colourless oil (61.0 mg, 91%); $[\alpha]_D^{27.4}$ = +16.9 (c = 1.04, $CHCl_3$), [lit [15] $[\alpha]_D^{25}$ = +21.6 (c = 1.04, $CHCl_3$)]; LCMS t_R 5.47 min, UV-trace only; ^{1}H NMR (600 MHz, $CDCl_3$) δ_H: 7.38–7.33 (m, 4H, H^8 and H^9), 7.30 (tt, J = 6.9, 1.6 Hz, 1H, H^{10}), 6.33 (d, J = 9.1 Hz, 1H, H^2), 4.54 (d, J = 12.1 Hz, 1H, H^6), 4.51 (d, J = 12.1 Hz, 1H, H^6), 3.40 (dd, J = 9.3, 6.3 Hz, 1H, H^5), 3.37 (dd, J = 9.3, 6.1 Hz, 1H, H^5), 2.84–2.77 (m, 1H, H^3), 1.08 (d, J = 6.9 Hz, 3H, H^4); ^{13}C NMR (150 Hz, $CDCl_3$) δ_C: 141.3 (CH, C^2), 138.4 (C, C^7), 128.5 (2 × CH, C^9), 127.7 (CH, C^{10}), 127.7 (2 × CH, C^8), 89.0 (CBr$_2$, C^1), 73.2 (CH$_2$, C^5), 73.1 (CH$_2$, C^6), 38.9 (CHCH$_3$, C^3), 16.0 (CH$_3$, C^4); IR (thin film) ν_{max}/cm^{-1} 3063.8 (w), 3029.4 (w), 2965.7 (w), 2930.6 (w), 2856.0 (w), 1614.5 (w), 1495.7 (w), 1474.7 (w), 1453.7 (w), 1358.8 (w), 1308.5 (w), 1237.2 (w), 1204.1 (w), 1155.4 (w), 1095.0 (m, br), 1028.1 (w), 922.8 (w), 905.4 (w),

[1] ^{1}H NMR: δ_H 1.51–1.47 (m, 1H, H^5 major) : 1.68–1.64 (m, 1H, H^5 minor)
[2] ^{1}H NMR: δ_H 2.57–2.50 (m, 1H, H^3 major) : 2.70–2.63 (m, 1H, H^3 minor)

872.3 (w), 842.9 (w), 782.0 (s), 733.3 (s), 695.9 (s); HRMS *m*/*z* (EI^+) found 253.0229 ($[M - Br]^+$, $C_{12}H_{14}OBr$ requires 253.0223), Δ = 2.6 ppm. The data for this compound is in agreement with that reported [15].

3,3-Dibromo-2-phenylacrylonitrile (Table 2, entry 4) [16]

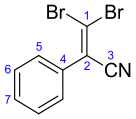

Isolated as a white crystalline solid (56.3 mg, 98%); mp 63–65 °C, [lit [16] 62–64 °C]; LCMS t_R 4.62 min, *m*/*z* (ESI^+) 287 ($[M + H]^+$); ^{1}H NMR (600 MHz, $CDCl_3$) δ_H: 7.50–7.47 (m, 2H, H^5), 7.46–7.43 (m, 3H, H^6 and H^7); ^{13}C NMR (150 MHz, $CDCl_3$) δ_C: 133.5 (*C*, C^4), 130.2 (*C*H, C^7), 129.1 (2 × *C*H, C^6), 128.7 (2 × *C*H, C^5), 122.8 (*C*, C^2), 117.2 (*C*N, C^3), 109.8 (*C*Br_2, C^1); IR (thin film) ν_{max}/cm^{-1} 3061.0 (w), 2924.8 (w), 2217.1 (w), 1886.0 (w), 1808.0 (w), 1758.9 (w), 1549.7 (w), 1485.0 (w), 1442.6 (w), 1390.9 (w), 1331.6 (w), 1284.2 (w), 1250.5 (w), 1183.9 (w), 1155.4 (w), 1106.6 (w), 1078.1 (w), 1057.0 (w), 1045.8 (w), 999.8 (w), 969.1 (w), 914.7 (w), 891.5 (w), 840.8 (s), 818.5 (m), 787.3 (m), 749.0 (s), 690.6 (s); HRMS *m*/*z* (EI^+) found 284.8785 ($[M]^+$, $C_9H_5NBr_2$ requires 284.8783), Δ = 0.7 ppm. The data for this compound is in agreement with that reported [16].

(4,4-Dibromo-3-methylbut-3-en-1-yn-1-yl)trimethylsilane (Table 2, entry 5) [17]

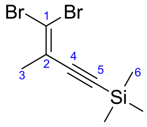

The general *gem*-dibromolefination reaction procedure was used, however the flow rate was decreased to 0.10 mL/min and the monolith was not used before this reaction. The output was collected for 2 h 30 min and the solvent removed in vacuo to give the desired product as a colourless oil (49.5 mg, 84%); LCMS R_t 5.69 min, *m*/*z* (ESI^+) 297 ($[M + H]^+$); ^{1}H NMR (600 MHz, $CDCl_3$) δ_H: 1.98 (s, 3H, H^3), 0.21 (s, 9H, H^6); ^{13}C NMR (150 MHz, $CDCl_3$) δ_C: 126.1 (*C*, C^2), 103.6 (*C*CSi, C^4), 102.5 (C*C*Si, C^5), 98.3 (*C*Br_2, C^1), 23.7 (CH_3, C^3), –0.2 (3 × Si(*C*H_3)$_3$, C^6); IR (thin film) ν_{max}/cm^{-1} 2959.3 (w), 2924.6 (w), 2853.9 (w), 2161.8 (w), 2120.1 (w), 1563.0 (w), 1409.0 (w), 1372.3 (w), 1249.9 (m), 1233.4 (w), 1026.5 (w), 897.6 (s), 833.6 (s), 758.9 (m), 700.0 (w); HRMS *m*/*z* (EI^+) found 293.9069 ($[M]^+$, $C_8H_{12}SiBr_2$ requires 293.9070), Δ = 0.2 ppm. The data for this compound is in agreement with that reported [17].

Flow synthesis of alkyl bromides in flow

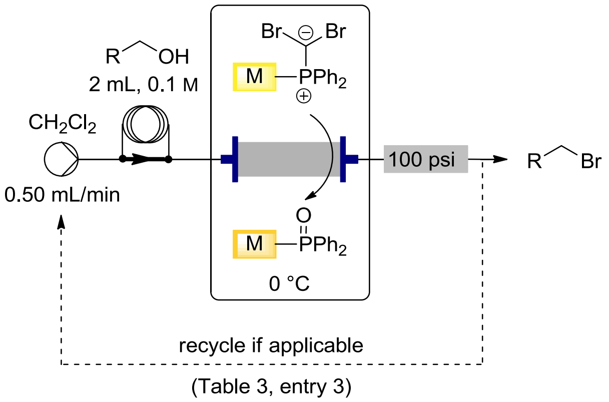

A stream of CH_2Cl_2 was pumped through the monolith loaded with carbon tetrabromide (kept at 0 °C) and then through a 100 psi back pressure regulator at 0.50 mL/min. A solution of the alcohol (0.2 mmol) in CH_2Cl_2 (2 mL) was prepared and injected into a 2 mL sample loop. The sample loop was switched inline and the output stream was collected for 1 h 15 min.

For Table 3, entry 3, the following modification was employed. The output from the flow setup was directed back into the CH_2Cl_2 stock solution (40 mL). The reaction was monitored by TLC and upon consumption of starting material, CH_2Cl_2 was passed through the monolith for 45 min at 0.50 mL/min. This output was combined with the previous stock solution and the solvent in vacuo to give the desired product.

It was found that following complete consumption of the active species for performing the dibromolefination reaction, it was then possible to use this monolith for the Appel reaction. The monolith was found to have an approximate active species loading for the Appel reaction of 0.55 mmol and during these bromination reactions the monolith changed colour from dull dark yellow to off-white.

Analysis of the off-white region gave the following data (sample collected under an inert atmosphere):
Elemental analysis found C 77.01, H 6.11, P 5.26, Br 10.50% (number of equivalents of Br to P = 0.77:1); IR (thin film) ν_{max}/cm^{-1} 1595.9 (w), 1486.6 (w), 1436.6 (w), 1405.3 (w), 1191.2 (w), 1115.5 (w), 1119.7 (m), 1068.3 (w), 1013.0 (w), 991.0 (w), 907.7 (w), 899.8 (w), 826.2 (w), 794.7 (w), 741.2 (w), 723.1 (m), 694.4 (s).

All yields reported below were obtained *via* the above procedure.

8-Bromo-2,6-dimethyloct-2-ene (Table 3, entry 1) [18]

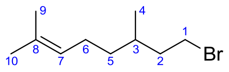

Isolated as a colourless oil (36.0 mg, 82%); LCMS t_R 5.66 min, UV-trace only; ^{1}H NMR (600 MHz, $CDCl_3$) δ_H: 5.09 (t, J = 7.0 Hz, 1H, H^7), 3.48–3.44 (m, 1H, H^1), 3.42–3.38 (m, 1H, H^1), 2.04–1.93 (m, 2H, H^6), 1.92–1.86 (m, 1H, H^2), 1.69 (s, 3H, H^{10}), 1.71–1.62 (m, 2H, H^2 and H^3), 1.61 (s, 3H, H^9), 1.37–1.32 (m, 1H, H^5), 1.21–1.15 (m, 1H, H^5), 0.90 (d, J = 6.5 Hz, 3H, H^4); ^{13}C NMR (150 MHz, $CDCl_3$) δ_C: 131.6 (*C*CH, C^8), 124.6 (C*C*H, C^7), 40.2 (*C*H_2, C^2), 36.7 (*C*H_2, C^5), 32.2 (*C*H_2Br, C^1), 31.5 (*C*H, C^3), 25.9 (*C*H_3, C^{10}), 25.5 (*C*H_2, C^6), 19.0 (*C*H_3, C^4), 17.8 (*C*H_3, C^9); IR (thin film) ν_{max}/cm^{-1} 2964.2 (s), 2915.7 (s), 2871.8 (m), 1742.5 (w), 1451.4 (s), 1377.9 (s), 1266.8 (m), 1215.6 (m), 1105.9 (w), 1022.7 (w), 985.2 (w), 830.7 (m), 753.9 (w); HRMS *m*/*z* (EI^+) found 218.0673 ($[M]^+$, $C_{10}H_{19}$Br requires 218.0665), Δ = 3.3 ppm. The data for this compound is in agreement with that reported [18].

3-(2-Bromoethyl)-1*H*-indole (Table 3, entry 2) [19]

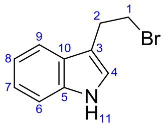

Output collected for 2 hrs. Isolated as a white crystalline solid (42.4 mg, 95%); mp 90–93 °C, [lit [19] 96–97 °C]; LCMS t_R 4.85 min, *m*/*z* (ESI^+) 145 ($[M – Br + H]^+$); ^{1}H NMR (600 MHz, $CDCl_3$) δ_H: 8.02 (br s, 1H, H^{11}), 7.61 (d, J = 7.9 Hz, 1H, H^9), 7.38 (d, J = 8.1 Hz, 1H, H^6), 7.22 (dt, J = 7.6, 0.6 Hz, 1H, H^7), 7.16 (dt, J = 7.6, 0.6 Hz, 1H, H^8), 7.10 (s, 1H, H^4), 3.65 (t, J = 7.7 Hz, 2H, H^1), 3.35 (t, J = 7.7 Hz, 2H, H^2); ^{13}C NMR (150 MHz, $CDCl_3$) δ_C: 136.3 (*C*, C^5), 126.1 (*C*, C^{10}), 122.4 (*C*H, C^7), 122.4 (*C*H, C^4), 119.7 (*C*H, C^8), 118.6 (*C*H, C^9), 113.7 (*C*CH_2, C^3), 111.4 (*C*H, C^6), 33.0 (*C*H_2Br, C^1), 29.5 (*C*H_2, C^2); IR (thin film) ν_{max}/cm^{-1} 3393.8 (m), 3048.9 (w), 2971.4 (w), 2938.2 (w), 2852.8 (w), 1702.1 (w), 1604.2 (w), 1549.9 (w), 1488.2 (w), 1455.7 (m), 1443.6 (w), 1421.5 (w), 1351.2 (w), 1340.5 (w), 1321.7 (w), 1297.5 (w), 1275.1 (w), 1241.8 (m), 1207.0 (m), 1153.7 (w), 1131.5 (w), 1114.5 (w), 1091.1 (m), 1058.4 (w), 1025.2 (w), 1007.5 (m), 962.1 (w), 933.3 (w), 876.5 (w), 850.7 (w), 823.7 (w), 770.7 (w), 750.8 (s), 654.3 (m); HRMS *m*/*z* (EI^+) found 144.0814 ($[M − Br]^+$, $C_{10}H_{10}$N requires 144.0808), Δ = 4.3 ppm. The data for this compound is in agreement with that reported [19].

(*Z*)-Methyl 2-(bromomethyl)-3-(dimethyl(phenyl)silyl)acrylate (Table 3, entry 3) [20]

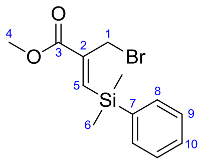

The solution was recycled through the monolith for 14 h 30 min. Isolated as a pale orange oil (56.1 mg, 90%); R_f = 0.86 (PE:Et_2O, 1:1); LCMS t_R 5.28 min, *m*/*z* (ESI^+) 220 ($[M - Br - CH_3 + H]^+$); ^1H NMR (600 MHz, $CDCl_3$) δ_H: 7.55 (dd, *J* = 7.6, 1.6 Hz, 2H, H^8), 7.42–7.38 (m, 3H, H^9 and H^{10}), 7.23 (s, 1H, H^5), 4.13 (s, 2H, H^1), 3.81 (s, 3H, H^4), 0.53 (s, 6H, H^6); ^{13}C NMR (150 MHz, $CDCl_3$) δ_C: 165.7 (*C*O, C^3), 145.3 (C*C*H, C^5), 143.9 (*C*CH, C^2), 136.5 (Si*C*, C^7), 133.9 (2 × *C*H, C^8), 129.9 (*C*H, C^{10}), 128.3 (2 × *C*H, C^9), 52.6 (OCH_3, C^4), 28.3 (CH_2Br, C^1), –1.9 (2 × SiCH_3, C^6); IR (thin film) ν_{max}/cm^{-1} = 3070.2 (w), 3049.2 (w), 3000.2 (w), 2953.2 (w), 1720.1 (s), 1600.5 (w), 1487.9 (w), 1428.2 (m), 1336.7 (w), 1233.4 (s), 1208.2 (m), 1152.2 (m), 1113.6 (m), 1064.1 (m), 998.4 (w), 986.4 (w), 968.9 (w), 888.1 (w), 815.6 (s), 782.5 (s), 731.2 (s), 698.6 (s), 654.5 (m); HRMS *m*/*z* (EI^+) found 296.9953 ($[M - CH_3]^+$, $C_{12}H_{14}O_2SiBr$ requires 296.9941), Δ = 3.93 ppm. The data for this compound is in agreement with that reported [20].

References

1. Pangborn, A. B.; Giardello, M. A.; Grubbs, R. H.; Rosen, R. K.; Timmers, F. J. *Organometallics* **1996,** *15,* 1518–1520.
2. Gottlieb, H. E.; Kotlyar, V.; Nudelman, A. *J. Org. Chem.* **1997,** *62,* 7512–7515.
3. Farrugia, L. J. *J. Appl. Cryst.* **1997,** *30,* 565–565.
4. Barton, T. J.; Groh, B. L. *J. Org. Chem.* **1985,** *50,* 158–166.
5. Rao, M. L. N.; Jadhav, D. N.; Dasgupta, P. *Org. Lett.* **2010,** *12,* 2048–2051.
6. Huh, D. H.; Jeong, J. S.; Lee, H. B.; Ryu, H.; Kim, Y. G. *Tetrahedron* **2002,** *58,* 9925–9932.
7. Berciano, B. P.; Lebrequier, S.; Besselièvre, F.; Piguel, S. *Org. Lett.* **2010,** *12,* 4038–4041.
8. Brown, G. R.; Clarke, D. S.; Foubister, A. J.; Freeman, S.; Harrison, P. J.; Johnson, M. C.; Mallion, K. B.; McCormick, J.; McTaggart, F.; Reid, A. C.; Smith, G. J.; Taylor, M. J. *J. Med. Chem.* **1996,** *39,* 2971–2979.
9. Cariou, K.; Mainetti, E.; Fensterbank, L. ; Malacria, M. *Tetrahedron* **2004,** *60,* 9745–9755.
10. Métay, E.; Hu, Q.; Negishi, E. *Org. Lett.* **2006,** *8,* 5773–5776.
11. Bach, P.; Nilsson, K.; Svensson, T.; Bauer, U. ; Hammerland, L. G.; Peterson, A.; Wållberg, A.; Osterlund, K.; Karis, D.; Boije, M.; Wensbo, D. *Bioorg. Med. Chem. Lett.* **2006,** *16,* 4788–4791.
12. Beny, J. P.; Dhawan, S. N.; Kagan, J.; Sundlass, S. *J. Org. Chem.* **1982,** *47,* 2201–2204.
13. Lok, W.; Ward, A. *Aust. J. Chem.* **1978,** *31,* 617–625.
14. Leyva, A.; Blum, F. E.; Hewitt, P. R.; Ley, S. V. *Tetrahedron* **2008,** *64,* 2348–2358.
15. Sneddon, H. F.; Gaunt, M. J.; Ley, S. V. *Org. Lett.* **2003,** *5,* 1147–1150.
16. Zhao, Z.; Li, Z. *Eur. J. Org. Chem.* **2010,** 5460–5463.
17. Robertson, J.; Naud, S. *Org. Lett.* **2008,** *10,* 5445–5448.
18. Flachsbarth, B.; Fritzsche, M.; Weldon, P. J.; Schulz, S. *Chem. Biodiversity* **2009,** *6,* 1–37.
19. Bowman, W. R.; Elsegood, M. R. J.; Stein, T.; Weaver, G. W. *Org. Biomol. Chem.* **2007,** *5,* 103–113.
20. Kolb, H. C.; Ley, S. V.; Slawin, A. M. Z.; Williams, D. J. *J. Chem. Soc., Perkin Trans. 1,* **1992,** 2735–2762.
